# 

**Published:** 1891-11-14

**Authors:** 


PRICE ONE PENNY.

No. 268, Vol. XI.—November 14th, 1891.

[Registered at the General Post Office as a Newspaper for transmission in the United Kingdom and Abroad.]

WITH EXTRA NURSING SUPPLEMENT.

Per Annum, 6s. 6d., post free.

Monthly Parts, 6d.; post free, 8d.

Ditto per Annum, 8s., post free.

### THE HOSPITAL

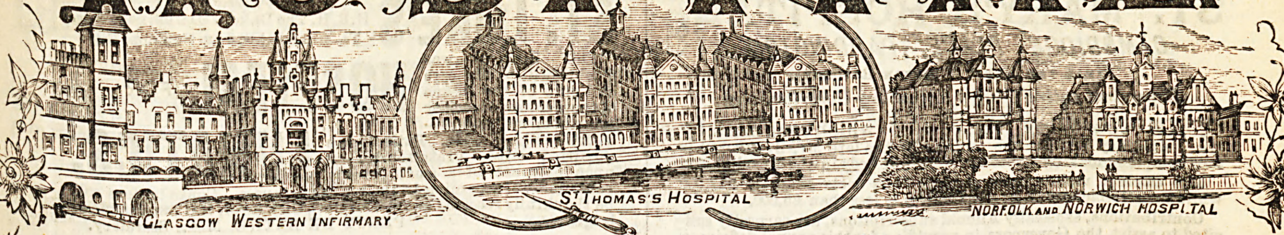

A WEEKLY JOURNAL OF

### Science, Medicine, Nursing, and Philanthropy.

SATURDAY, NOVEMBER 14th, 1891.

**The Antivivisection Memorial** . . . . . . 73

**Passing Topics.**—The Proposed Russian Famine Fund . . 74

**The Doctor's Armchair.**—Old Doctors or New? . . . 75

**Hospitals of the North-West** I.—Winnepeg and Medicine Hat . . . . . . . 76

**Everybody's Page** . . . . . . . 77

**Notes and News** . . . . . . . 78

**General Charities** . . . . . . . 79

**Scraps and Gleanings** . . . . . . . 79

**Annotations.**—"That Poor Worm, the Doctor"—An Undervalued Profession—An Important Hospital Experiment . 80

**The Practitioner's Mirror and Retrospect—**

MEDICINE.—New Methods of Treatment in Phthisis, VII.—The Lannelongue Treatment of Tubercular Joints and Bones 81

OPHTHALMOLOGY.—Mydriatics . . . . . . 81

NEW DRUGS, APPLIANCES, AND THINGS MEDICAL.—Addendum . . . . . . . 82

**Turning Over New Leaves.**—The Medical Digest . . 83

**Short Trips for Short Holidays.** II.—A Peep at Russia, and Home by Sweden (*concluded*) . . . . . 83

**The Student of Medicine.**—Students' Medical Societies—Appointments—Vacancies

EXTRA SUPPLEMENT.—THE NURSING MIRROR.

**En Passant.**—York Road Hospital—Glasgow Nurses—Clergy Confer about Nurses—Ipswich Nurses' Home—Workhouse Nursing—Irish Lunatics—Short Items . . . xxxvii

**A National Pension Fund for American Nurses** . xxxviii

**On the Nursing of Sick Children** . . . . . xxxix

**Notes from Australia** . . . . . . xxxix

**For Reading to the Sick.**—Polishing . . . . xxxix

**Appointments** . . . . . . . . xxxix

**Presentations** . . . . . . . . xlii

**Everybody's Opinion.**—Local High Temperature—Nursing Arrangements at the Metropolitan Hospital—Proposed Memorial to the late Miss Freeman—Nurses as Patients . . . . . . . . xli

**Christmas Competitions** . . . . . . xli

**Notes and Queries** . . . . . . . xli

**Off Duty.**—Bruce (*concluded*) . . . . . xlii

**A Bed for a Sick Nurse** . . . . . . xlii

**Amusements and Relaxation** . . . . . xlii

